# The Role of VibraPlus on Fatigue in Multiple Sclerosis Patients: A Randomized Controlled Trial

**DOI:** 10.3390/jcm14113990

**Published:** 2025-06-05

**Authors:** Caterina Formica, Desirée Latella, Lilla Bonanno, Antonino Lombardo Facciale, Giuseppe Paladina, Antonino Leo, Luca Pergolizzi, Bartolo Fonti, Angelo Quartarone, Roberta Cellini, Rocco Salvatore Calabrò

**Affiliations:** IRCCS Centro Neurolesi Bonino Pulejo, 98124 Messina, Italy; katia.formica@irccsme.it (C.F.); lilla.bonanno@irccsme.it (L.B.); antonino.lombardo@irccsme.it (A.L.F.); giuseppe.paladina@irccsme.it (G.P.); antonino.leo@irccsme.it (A.L.); luca.pergolizzi@irccsme.it (L.P.); bartolo.fonti@irccsme.it (B.F.); angelo.quartarone@irccsme.it (A.Q.); roberta.cellini@irccsme.it (R.C.); roccos.calabro@irccsme.it (R.S.C.)

**Keywords:** rehabilitation, vibration training, multiple sclerosis, quality of life, motor outcome

## Abstract

**Background and Objective:** Fatigue represents a hallmark symptom in Multiple Sclerosis (MS), but its diagnosis and clinical evaluation is difficult because it is described as a subjective feeling of exhausted physical and mental sensation. Studies have also shown that approaches based on assisted therapies and robotics, as well as the use of vibration, which are used to improve sensory integration, reduce fatigue. The primary outcome in this study is to evaluate the effects of the application of focal vibrations on the reduction in fatigue, muscle strength, and endurance in MS patients with moderate disability. The secondary outcome is to assess the effects on quality of life, cognitive status, and mood. **Methods:** We enrolled 40 MS patients. The study was designed as a parallel randomized controlled trial: 20 patients were assigned to the experimental group (EG), who received vibration training, and 20 to the control group (CG), who received traditional physical exercise. **Results:** We found significant differences in the EG in fatigue, motor, and cognitive outcome and improvement of some aspects of quality of life (QoL). There are correlations between perceived multidimensional fatigue and cadence, step length, and health quality of life composite. **Conclusions:** Our study demonstrated the potential effectiveness of vibration training in balance, walking endurance, and reduction in the risk of falls in patients with Multiple Sclerosis. In addition, we added evidence about fatigue, non-motor outcomes, in particular promoting mental and physical QoL and individual life satisfaction. The name of the registry is clinicaltrial.gov; the number of registration id NCT05783999; and the date of registration is 14 March 2023.

## 1. Introduction

In the complex realm of neurological disorders, Multiple Sclerosis (MS) represents one of the main causes of neurological disability among young and middle-aged adults. MS affects over 2.8 million people globally and is most frequent in women (women–men ratio: 2–4:1) [1,2]. MS is an autoimmune disease characterized by demyelination of the sensory and motor nerves in the central nervous system [3]. The progression of the disease is characterized by stable periods interrupted by phases with the activity of the disease (relapsing-remitting form) or by a continuous progression such as in primary and secondary forms of MS [4]. The symptoms of MS includes muscle weakness, balance instability, spasticity, fatigue, cognitive deficits, depressive symptoms, and sensory disturbances [5]. There is no unique treatment for this symptomatology. With the exception of the use of painkillers, muscle relaxants and Fampridine may have beneficial effects on walking [6,7,8]. Consequently, the use of non-pharmacological treatments could be warranted. The progressive resistance training provided is a promising intervention for muscle strength, while there are discordant results about its efficacy for walking and balance [9]. Fatigue represents a hallmark symptom in MS, and its diagnosis and clinical evaluation is difficult because it is described as a subjective feeling of exhausted physical and mental sensation. It affects two-thirds of MS patients and has been strictly associated with health-related quality of life (QoL) [10]. Fatigue can appear in the early stages of MS, even as a prodromal symptom of the disease [11]. MS fatigue is often interpreted as depression, or poor concentration, creating considerable discomfort [12]. It is noteworthy that fatigue interferes with physical and mental activities and contributes to worsening existing disabilities and negatively interfering with daily living and social and working life activities [13]. The etiology of fatigue is unclear: central and peripheral mechanisms are involved, which may be related with some peculiar disease features, such as inflammation, demyelination, and neurodegeneration [14]. Inflammatory mediators such as pro-inflammatory cytokines like tumor necrosis factor (TNF) and Interleukin-6 (IL-6) have also been proposed to be involved in the pathophysiology of fatigue [14]. The evidence provided that physical exercise had a therapeutic effect on inflammatory diseases. Therefore, intense resistance training was well tolerated and contributed to the reduction in self-reported fatigue [15,16]. Englund et al. [17] conducted a study of a 12-week supervised high intensity resistance training (HIRT) program in patients with relapsing remitting MS (RRMS), indicating clinically relevant improvements in fatigue, a decrease in anxiety and depression scores, improved QoL, and reduced levels of TNF in blood [17,18]. Other studies evaluated the effectiveness of sensory integration-based interventions on fatigue. These latter interventions had significant beneficial effects on MS patients; in particular, they led to improved balance and quality of life [19,20,21]. Studies have also shown that approaches based on assisted therapies and robotics, as well as the use of vibration, can also be used to improve sensory integration and reduce fatigue [22,23]. In particular, vibrations have been proposed as a complementary technique in the treatment and rehabilitation of neurologic disorders. Some studies showed that both whole-body vibration (WBV) and focal muscle vibration (FMV) contributed to reduce spasticity and improve gait, balance, and motor function in stroke patients. By contrast, vibration therapy seems to be unable to reduce spasticity in Multiple Sclerosis and cerebral palsy [24]. On the other hand, it was demonstrated that vibration has a beneficial effect in MS patients compared to patients that used physical exercise alone [25]. The mechanism underlining the vibration treatment was unclear in neurologic patients and is still under investigation, but some authors have suggested that vibration improves muscle contraction and increases muscle strength pain and balance [26,27]. Different clinical factors, such as a patient’s type of MS, EDSS score, and cognitive status, could interfere with the success of the treatment.

The primary outcome in this study is to evaluate the effects of the application of focal vibrations on the reduction in fatigue, muscle strength, and endurance in MS patients with moderate disability. The secondary outcome is to assess the effects on quality of life, cognitive status, and mood.

## 2. Materials and Methods

### 2.1. Study Design and Population

**Figure 1:**
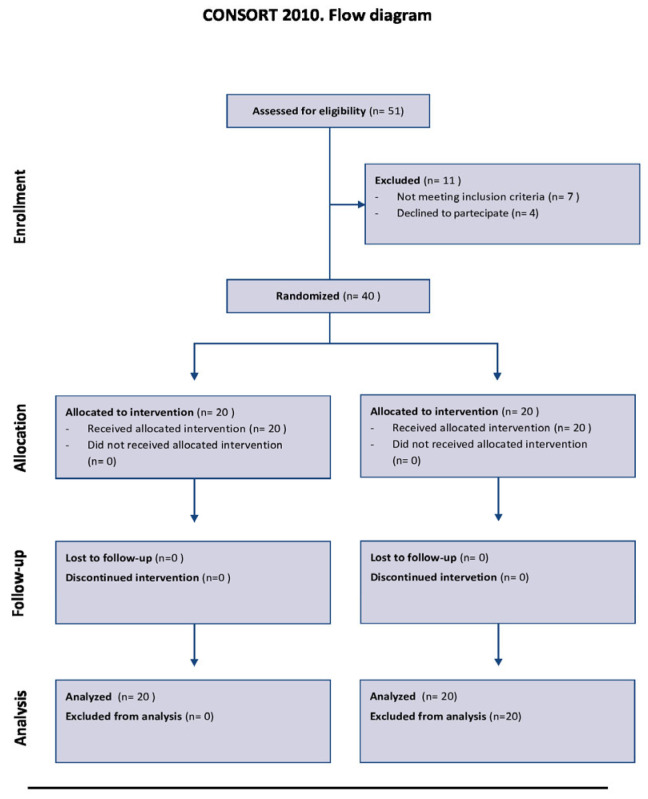
CONSORT Flow Diagram 2010, [28]. Moher, D.; Hopewell, S.; Schulz, K.F.; Montori, V.; Gøtzsche, P.C.; Devereaux, P.J.; Elbourne, D.; Egger, M.; Altman, D.G. (2012) [28]. CONSORT 2010 Spiegazione ed Elaborazione: linee guida aggiornate per il reporting di trial randomizzati a gruppi paralleli. Evidence, 4, e1000024.

We included 40 patients affected by relapsing remitting Multiple Sclerosis who had an Expanded Disability Status Scale (EDSS) score between: 3.5 to 6. Patients were enrolled from the Multiple Sclerosis Outpatient Clinic of the IRCCS Centro Neurolesi “Bonino-Pulejo” in Messina from March 2023 to March 2025 (name of the registry: clinicaltrial.gov; number of registration: NCT05783999; date of registration: 14 March 2023). The study was designed as a parallel randomized controlled trial and 20 patients were assigned to the experimental group (EG), who received vibration training, and 20 to the control group (CG), who received traditional physical exercise (Figure 1). We employed purposive sampling to select participants for this study. Purposive sampling is a non-probability sampling technique used when specific criteria are required to ensure the sample aligns with the research objectives. Purposive sampling was chosen to ensure that all participants had similar characteristics relevant to the study’s aims, particularly the diagnosis of MS. Randomization was performed using simple randomization from random.org. Outcome assessors were blinded to group assignment (single-blind design), although participants and treating therapists were necessarily aware of allocation.

Forty patients were included according to inclusion criteria as follows: diagnosis of MS; evident fatigue symptoms; age between 20 and 55; and EDSS score between 3 and 6. We excluded patients with secondary form MS; presence of psychiatric disorders; presence of pacemaker or other subcutaneous implants; and presence of musculoskeletal disorders.

The Local Ethics Committee of IRCCS Centro Neurolesi “Bonino Pulejo” approved the study (registration n. U74/21), and participants gave their signed informed consent.

### 2.2. Procedures and Device

Each EG participant underwent 30 min of focal MV to quadriceps and gastrocnemius bellies—using the Vibra Plus [29] with 2 cm^2^ silicone-capped probes—applied during upright stance (quadriceps) or passive dorsiflexion (gastrocnemius). Frequency was 100 Hz, amplitude individually set at 0.2–0.5 mm (just below illusion threshold, a-peak = (2πf)^2^·A), and delivered in five 60 s bouts with 30 s rest (30 min/session), three times/week for seven weeks under single-blind conditions. Focal MV of the quadriceps enhances motor-unit recruitment and fatigue resistance—key for stance and push-off—and induces reflex inhibition of hamstring antagonists, reducing knee stiffness. Similarly, gastrocnemius MV modulates spinal reflexes to decrease calf spasticity and, when applied in passive stretch, may reset proprioception and lower pathological tone. Vibra plus represents an innovative and non-invasive system for the treatment of muscular and neuromuscular conditions. It harnesses the power of meccano-acoustic waves to create therapeutic vibrations. These vibrations could trigger adaptive responses in neuro-muscular and musculoskeletal systems, influencing metabolism and neurophysiology. The intended therapeutic effect lies in its ability to stimulate muscles and sensory receptors, potentially enhancing muscle strength, coordination, and balance, which can help reduce the effort required for daily activities and thus decrease perceived fatigue. Additionally, vibration may improve blood circulation and lymphatic drainage, promoting better oxygen delivery to tissues and more efficient removal of metabolic waste, leading to increased energy levels. Some studies also suggest a potential stimulatory effect on the central nervous system, which could enhance neuromuscular communication and support neuroplasticity [30]. The initial exploration of vibrations in medicine start in 1949, but only in recent years has the technological progression been provided to exploit their therapeutic power. The device is equipped with applicators (domes) of three different sizes; these applicators are applicated to the skin using biocompatible silicone discs, during treatment. Vibra accelerates functional recovery by enhancing proprioception, muscle tone, physical endurance, and muscle coordination. Vibrations have a muscle-relaxing effect on spasticity, proving especially effective in the field of neurorehabilitation. Vibra effectively reduces muscle tone resulting from central nervous system lesions by leveraging the neurophysiological mechanism of reciprocal inhibition. The afferent signals, activated by vibrations, transmit mechano-sonic information from muscles to spinal nerve circuits, where initial therapeutic interactions occur. These overlapping signals results in the “reprogramming of motor engrams”, leading to improvements in proprioception, performance, and re-education, accompanied by optimized muscle tone, increased endurance, and improved muscle coordination. Additionally, the vibration stimuli induce pain control without releasing endorphins. A combined approach with physical activity could improve metabolic effects, enhancing functional recovery. The CG was submitted to traditional physical treatment based on aerobic exercises from slow to moderate intensity; cyclette and tapis roulant were used3 times a week for 30 min for each session, providing evidence for the improvement of cardiorespiratory activity and reduction in fatigue. Resistance training activity based on muscle-strengthening exercises was performed 2 times a week for 45 min each session to increase resistance to muscle fatigue and improve functional endurance. Treatment for conservation of energy and pacing was based on the education of the patient to distribute daily activities (“pacing”), alternating phases of activity and rest, and use of aids (wheelchairs, lifting devices) to minimize energy expenditure.

### 2.3. Outcome Measures

The physiotherapy assessment was carried out using the following tools:

- Berg Balance Scale (BBS) consists of 14 mobility tasks, with tasks varying in levels of difficulty. Tasks are divided into 3 domains, namely seated balance, standing balance, and dynamic balance. Each activity is assessed on a Likert scale of 5 points with a maximum score of 56. A total score below 45 is associated with a higher risk of falls [31].
- The 6-Minute Walk Test (6MWT) assesses the distance covered in 6 min and provides information about endurance and cardiorespiratory function and evaluates the response to therapeutic treatments [32].
- The Modified Borg Dyspnea Scale (MBS) is a numerical score rated from 0 to 10 used to measure dyspnea as reported by the patient during intense exercise [33].
- Modified Timed Up and Go (TUG) with bilateral turns provides an observational approach to gait assessment and can help to predict the risk of falls. The test assesses the time to rise from a chair, walk 3 m, turn around, and then sit down again. It has been suggested that a cut-off point of 13.5 s may identify individuals with an increased risk of falls [34].
- The 10-Meter Walk Test is a performance measure used to assess gait speed in meters per second over a short distance. The total time taken to walk 6 m is recorded [35].
- The Expanded Disability Status Scale (EDSS) measures the level of disability in MS patients with a range scale from 0 to 10. The initial levels from 1.0 to 4.5 refer to individuals with a high degree of walking ability, from 5.0 to 9.5, pertain to the loss of walking ability [36].
- The Fatigue Severity Scale (FSS) assesses the impact of perceived fatigue on a patient. The instrument consists of Likert scales from 1 to 7 (1 completely agree, 7 completely disagree) [37].
- The Modified Fatigue Impact Scale (MFIS) assesses perceived fatigue and the impact on physical, social, and cognitive levels [38].
- The Fatigue Scale for Motor and Cognitive Functions (FSMC) is a scale composed of 20 items, with 10 items related to cognitive fatigue (FSMC cog) and 10 items related to motor fatigue (FSMC mot). The instrument consists of Likert scales from 1 to 5 points. The total possible score ranges from 20 to 100 points. A total score of ≥43 is classified as mild fatigue, ≥53 as moderate fatigue, and ≥63 as severe fatigue [39].

The neuropsychological assessment was carried out as follows:

The Brief Repeatable Battery of Neuropsychological Tests (BRB-N) are a sensitive measure of cognitive impairment in multiple sclerosis (MS) patients. It consists of the Selective Reminding Test, the 10/36 Spatial Recall Test, the Symbol Digit Modalities Test, the Paced Auditory Serial Addition Test, and the Word List Generation Test [40].

Multiple Sclerosis Quality of Life (MSQOL-54) is a disease-specific instrument used to measure the quality of life of an MS patient with 18 disease-specific dimensions, which measure the anxiety provoked by one’s health status (four items), sexual functioning (four items), satisfaction with sex life (one item), overall quality of life (two items), cognitive functioning (four items), energy (one item), pain (one item), and social functioning (one item). The instrument consists of Likert scales and multiple-choice items [41].

### 2.4. Gait Analysis

Participants of the EG also performed motion analysis at a specific laboratory located within the “BTS Gait Lab” at baseline (T0) and after 20 sessions of treatment (T1). Gait analysis was performed with 8 infrared cameras, 4 sensitized platforms, and 8 wireless electromyographic probes. The selected Gait Analysis Protocol for this study is “DAVIS: multifactorial gait analysis”. The optoelectronic system measures the coordinates of markers placed on the patient’s body, and suitable software uses these coordinates to calculate the angles of flexion/extension, abduction/adduction, and external/internal rotation of the hip, knee, and ankle joints. These markers are placed at the following reference points: acromion, C7, anterior superior iliac spine (ASIS), L5, greater trochanter of the femur, posterior femoral condyle, head of the fibula, lateral malleolus, calcaneus, and 5th metatarsal head. Additionally, four markers are attached to a bar at a predefined distance, positioned at mid-thigh and mid-calf relative to the sagittal plane. The electromyographic signals are revealed with probes that are applied at the level of the tibialis anterior, lateral gastrocnemius, rectus femoris, and semitendinosus muscles of both lower limbs. During the first phase, referred to as “standing”, the subject is asked to reach a specific platform and maintain an orthostatic position for 6 s. In the second phase, referred to as “Walking”, the subject is instructed to walk at a self-perceived normal speed and cadence while crossing the platforms. The final report reveals data related to kinematics (angles of flexion/extension, abduction/adduction, and external/internal rotation of the major hip, knee, ankle, and pelvic joints), dynamics (moments and powers at the hip, knee, and ankle joints), and electromyographic (muscle activation and deactivation) parameters.

## 3. Statistical Analysis

Descriptive analysis was reported for demographic and clinical variables. Continuous variables were expressed as mean ± standard deviation, whereas categorical variables were in frequencies and percentages. The numerical data were presented in median, and first-third quartile in no normal distribution. Unpaired Student’s t-test or the Mann–Whitney U test was used for inter-group analysis, while paired Student’s t-test or Wilcoxon signed rank test was used for intra-group analysis.

Spearman’s rank correlation was applied to investigate potential relationships between QoL and MFIS, FSMC, FSS, EDSS, and VAS. Analyses were performed using an open source R4.2.2 software package. A 95% of confidence level was set with a 5% alpha error. Statistical significance was set at *p* < 0.05.

## 4. Results

### 4.1. Motor Outcome

We found significant differences between the EG and CG in some motor scale scores (Table 1), in particular at T0 in TUG Right (*p* = 0.04), TUG Left (*p* = 0.02), MFIS (*p* = 0.04), EDSS (*p* = 0.01), and VAS Fatigue (*p* = 0.02), and at T1 in EDSS (*p* = 0.01). Significant differences between T0 and T1 in the EG were found via intra-group analysis in BERG (*p* < 0.001), TUG Right (*p* < 0.001) and TUG Left (*p* < 0.001), 6 min (*p* < 0.001), and VAS fatigue (*p* = 0.001), while no significance differences were found in the CG.

### 4.2. Gait Analysis Outcome

**Figure 2:**
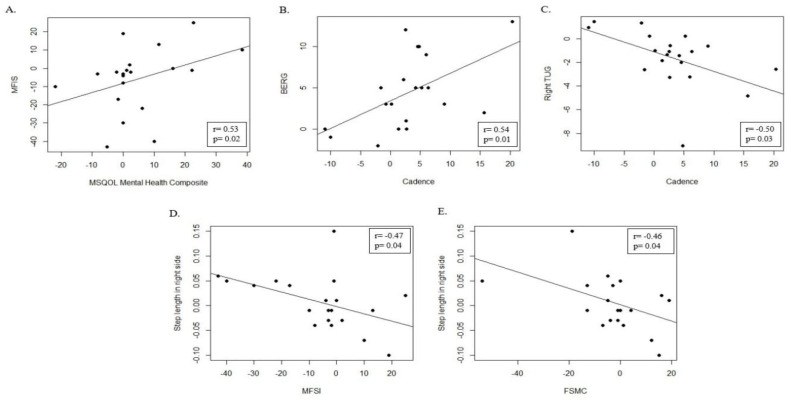
Correlation in the experimental group. (**A**) Scatter plot of MFIS score and MSQOL Mental Health Composite. (**B**) Scatter plot of BERG score and cadence. (**C**) Scatter plot of right TUG score and cadence. (**D**) Scatter plot of step length in right side score and MFSI. (**E**) Scatter plot of step length in right side score and FSMC.

Intra-group analysis did not show significant differences in gait analysis scores. No significant differences between T0 and T1 in cadence (*p* = 0.07), breadth (*p* = 0.74), step length in the right side (*p* = 0.60), and in step length in the left side (*p* = 0.84) were found. Moreover, we found a significant positive correlation between cadence and BERG (r = 0.54; *p* = 0.01) (Figure 2B) while there was a significant negative correlation between cadence and right TUG (r = −0.50; *p* = 0.03) (Figure 2C). Another significant negative correlation was between step length in the right side with MFSI (r = −0.47; *p* = 0.04) (Figure 2D) and FSMC (r = −0.46; *p* = 0.04) (Figure 2E). Finally, a trend correlation was found between step length in the left side and FSS (r = −0.40; *p* = 0.08).

### 4.3. Cognitive Outcome (BRB-N)

Inter-group analysis showed significant differences between the experimental and control groups in some BRB-N sub-item scores (Table 2), in particular, at T0 in SPART (*p* = 0.04) and SRT-D (*p* = 0.04). In the experimental group, intra-group analysis showed significant difference between T0 and T1 in SRT-LTS (*p* = 0.002), SRT-CLTR (*p* = 0.04), SPART (*p* = 0.03), and SDMT (*p* = 0.02), while in the control group it was only in PASAT-2 (*p* = 0.03). No significance correlation was found in the experimental group.

### 4.4. Quality of Life Outcome (MSQOL-54)

The comparison between the EG and CG in the MSQOL-54 test (Table 3) showed significant differences at T0 in Health Perception (*p* = 0.01), Energy/Fatigue (*p* = 0.003), Sexual Functions (*p* = 0.003), Physical Health Distress (*p* = 0.03), and at T1 in total score MSQOL Physical Health Composite (*p* = 0.02), Sexual Functions (*p* = 0.004), and Cognitive Functions (*p* = 0.04). Intra-group analysis highlighted no significative differences between T0 and T1 in the EG, while in the CG a difference was found in Energy/Fatigue (*p* = 0.03). Correlation analysis, in the experimental group, showed a positive significant correlation between MFIS and total score MSQOL Mental Health Composite (r = 0.53; *p* = 0.02) (Figure 2A) and a trend between MFIS and Social Functions (r = 0.43; *p* = 0.06), FSMC and Sexual Functions (r = −0.44; *p* = 0.05), FSS and Physical Health Distress (r = 0.42; *p* = 0.06), and between VAS and Role Emotional Limitation (r = −0.40; *p* = 0.08).

## 5. Discussion

Rehabilitation is the better treatment strategy for the MS population to ease the burden of the motor symptoms and optimize the quality of life, promoting wellness and social, personal, and daily living activities [42,43]. One of the tools used in rehabilitation programs in neurological population is vibration therapy, which has potential benefits on muscle performance, mobility, proprioception, and less health distress [44,45]. In this study, we investigated the effects of focal vibration in fatigue, muscle strength, and endurance and their impact on quality of life and cognitive performance. We found that WBV had outcomes in motor function, cognition, and some aspects of QoL. In the EG, significant differences were found after vibration training, in intra-group analysis in BERG (*p* < 0.001), TUG Right (*p* < 0.001), and TUG Left (*p* < 0.001), in 6 min (*p* < 0.001) where balance and risk of falls were evaluated, and VAS fatigue (*p* = 0.001) about the perception of fatigue, while no significance differences were found in the CG (Table 1). A modified version of the Timed Up and Go (TUG) test was used for observational purposes, with the specific intent of preventing participants from consistently selecting their stronger side. A correlation between cadence, step length, and fatigue perceived were found (Figure 2C–E). Some literature studies demonstrated beneficial effects of WBV in fine motor coordination and functional mobility [46] in balance and walking endurance [47,48]. However, the evidence for the effects of vibration therapy on functional mobility, gait speed, fatigue, cognition, and quality of life remains unclear. A meta-analysis, investigating vibration training in motor and non-motor symptoms in MS, showed no significant results on fatigue compared to control group; also, there is less scientific evidence focused on WBV effects in mental and physical health [49]. In accordance with other studies, our findings suggested that intensive vibration training contributes to motor outcome improving balance, risk of falls, and fatigue in MS patients (Table 1). Few studies focused on the impact of vibration therapy on non-motor impairments in MS. Indeed, our results suggested that vibration had a beneficial effect in some cognitive functions such as long-term memory, visuo-spatial memory, and processing speed skill (Table 2), aligning with Su and Chang’s findings of a +2.3-point increase in MoCA score following a 12-week WBV regimen (Eurtronik S.r.l., 2024) [29]. Nonetheless, systematic reviews emphasize the need for larger, adequately powered RCTs using standardized neuropsychological batteries to confirm these preliminary benefits. Moreover, there is still little research investigating the effects of vibration training on the general health status, with discordant results [50,51], and most of these studies used only MSQOL-54 and MSIS-29 to assess the health-related quality of life. In our study, we used a detailed set of standardized tests to evaluate, in a specific way, all aspects of fatigue, motor, cognitive functions, and the impact on QoL. Moreover, our results suggest that vibration training can improve non-motor aspects of QoL. We found a positive correlation between MFIS fatigue scores and SF-36 mental health subscales, as well as positive trends in sexual function, social function, and role limitations due to emotional problems (Table 3), consistent with Krause et al.’s report of psychological benefits from prolonged WBV protocols in MS [52] and supporting the integration of WBV into a multimodal rehabilitation strategy. Our results suggested that vibration training could improve some non-motor aspects about QoL; in fact, we found a positive correlation between fatigue tests and mental health scores and a positive trend about fatigue score and sexual functions, social function, and role limitations due to emotional problems (Table 3). In addition, some aspects of gait, as well as step length, correlated with cognitive and motor fatigue for people with MS (Figure 2A). It is well known that MS patients have lower general QoL compared to other chronic disease populations [53]. Fatigue and restricted walking are the most important symptoms that limit MS patients from participating in social and familiar activities [54,55,56]. Consequently, the use of a multimodal approach to manage fatigue, combining psychological and physical features, was suggested. Our results added knowledge to the insufficient literature evidence about the importance of vibration treatment for reducing motor impairment, motor, and non-motor fatigue, as well as facilitating socialization and decreasing limitation due to physical and emotional problems, promoting a better perception of one’s own QoL. However, this study presented some limitations. First, the sample size is small and the results cannot be extended to the MS population; second, at baseline, the EG and CG were not homogeneous as well as in disability level; and third, the lack of gait analysis in the CG. Finally, there is the absence of follow-up to evaluate the effectiveness of vibration therapy because it had a significant value in clinical practice. Therefore, a larger-sample-sized RCTs with a more homogeneous population are needed to confirm these promising results.

### Limitations

This study has several important limitations that should be acknowledged. The small sample size limits statistical power and external validity to the broader MS population, so future multicenter trials with an a priori power calculation are needed to ensure adequate sample sizes and enable subgroup analyses by MS subtype. Baseline imbalances in EDSS scores between the experimental and control groups may have confounded our treatment effects; employing stratified randomization or covariate-adaptive allocation in future studies will help ensure group comparability at baseline. The absence of a sham or alternative control intervention prevents us from ruling out placebo or nonspecific device effects, highlighting the need for a sham-vibration arm with an identical setup but without mechanical oscillation to achieve a more rigorous single-blind design. Since gait analysis was performed only in the experimental group, we cannot directly compare spatiotemporal and kinematic outcomes across arms; future work should include identical motion-capture protocols in both study groups. Finally, the lack of follow-up beyond the eight-week intervention means the durability of motor and non-motor benefits remains unknown; extending follow-up assessments to 6 and 12 months post-intervention will be essential to determine whether observed improvements are sustained.

## 6. Conclusions

Our study demonstrated the potential effectiveness of vibration training in balance, walking endurance, and reduction in the risk of falls in patients with Multiple Sclerosis. In addition, we added evidence about fatigue, non-motor outcomes, in particular promoting mental and physical QoL and individual life satisfaction. Future directions should focus on long-term effectiveness and introduce vibration therapy in a rehabilitative multimodal approach.

## Figures and Tables

**Table 1:** Inter–intra analysis in motor outcome.

		EG	CG	*p*-Value
BERG	T0	42.8 ± 9.0	48.5 (39.3–54.0)	0.31
	T1	47.5 (43.8–52.2)	48.5 (39.3–54.0)	0.91
	*p*	0.0002 *	NA	
10M	T0	7.7 ± 3.3	6.1 (5.5–9.1)	0.69
	T1	6.0 (4.8–9.2)	6.1 (5.5–9.1)	0.34
	*p*	0.11	NA	
TUG Right	T0	14.3 ± 5.7	8.2 (7.6–12.5)	0.04 *
	T1	11.9 ± 4.2	8.2 (7.6–12.5)	0.15
	*p*	0.007 *	NA	
TUG Left	T0	14.8 ± 6.1	7.9 (7.1–12.1)	0.02 *
	T1	13.0 ± 5.6	7.9 (7.1–12.1)	0.09
	*p*	0.02 *	NA	
BORG	T0	3.4 ± 2.0	3.6 ± 3.0	0.83
	T1	3.0 (1.8–4.4)	3.6 ± 3.0	0.88
	*p*	0.94	NA	
MFIS	T0	51.9 ± 14.9	38.7 ± 23.4	0.04*
	T1	46.0 ± 17.5	40.5 ± 22.8	0.27
	*p*	0.15	0.33	
FSMC	T0	71.8 ± 17.0	66.4 ± 22.5	0.4
	T1	68.9 ± 18.2	66.4 ± 22.5	0.7
	*p*	0.41	NA	
FSS	T0	48.7 ± 10.8	41.6 ± 12.1	0.06
	T1	46.6 ± 10.7	41.6 ± 12.1	0.17
	*p*	0.23	NA	
6 MIN	T0	263.6 ± 115.4	351.0 (232.5–378.5)	0.2
	T1	285.9 ± 125.1	351.0 (232.5–378.5)	0.52
	*p*	0.006 *	0.37	
EDSS	T0	5.5 (4.4–6.0)	3.5 (3.5–4.1)	0.01 *
	T1	5.3 (4.4–5.6)	3.5 (3.5–4.1)	0.01 *
	*p*	0.59	1	
VAS fatigue	T0	7.7 ± 1.3	7.0 (6.0–7.0)	0.02 *
	T1	6.5 ± 1.90	7.0 (6.0–7.0)	0.73
	*p*	0.001 *	NA	

* *p* < 0.05. Data are presented as means ± standard deviations for normally distributed variables and medians (first and third quartiles) for non-normally distributed variables.

**Table 2:** Inter–intra analyses in cognitive outcome.

		EG	CG	*p*-Value
SRT-LTS	T0	26.2 ± 15.5	28.4 ± 10.7	0.61
	T1	34.7 ± 16.6	28.1 ± 13.6	0.18
	*p*	0.002 *	0.93	
SRT-CLTR	T0	21.4 ± 16.4	23.6 ± 9.6	0.62
	T1	26.7 ± 16.7	23.7 ± 5.5	0.44
	*p*	0.04 *	0.96	
SPART	T0	15.9 ± 5.0	19.0 ± 4.3	0.04 *
	T1	18.1 ± 6.1	19.7 ± 4.5	0.35
	*p*	0.03 *	0.37	
SDMT	T0	33.0 ± 12.4	34.9 ± 13.4	0.64
	T1	36.3 ± 13.3	35.0 ± 12.7	0.75
	*p*	0.02 *	0.95	
PASAT-3	T0	31.2 ± 13.3	40.0 (16.4–46.5)	0.68
	T1	36.6 ± 16.8	40.0 (16.4–46.5)	0.6
	*p*	0.11	0.18	
PASAT-2	T0	27.2 ± 15.4	25.9 ± 15.1	0.78
	T1	31.9 ± 14.8	26.5 ± 14.2	0.25
	*p*	0.07	0.03 *	
SRT-D	T0	6.1 ± 3.0	7.8 ± 2.2	0.04 *
	T1	6.6 ± 3.0	7.9 ± 2.0	0.12
	*p*	0.22	0.81	
SPART-D	T0	5.9 ± 2.6	6.9 ± 2.2	0.19
	T1	7.2 (3.9–9.04)	7.1 ± 1.9	0.63
	*p*	0.05	0.1	
WLG	T0	21.1 ± 5.9	20.7 ± 5.9	0.84
	T1	21.8 ± 5.8	19.9 (18.9–25.1)	0.61
	*p*	0.34	0.37	

* *p* < 0.05.

**Table 3:** Inter–intra analyses in quality of life.

		EG	CG	*p*-Value
MSQOL Physical Composite	T0	90.8 ± 42.1	81.8 (53.7–91.3)	0.32
	T1	95.3 ± 37.5	74.1 (50.0–82.8)	0.02 *
	*p*	0.47	0.06	
Physical Function	T0	6.4 ± 4.7	9.8 (4.3–13.8)	1
	T1	8.4 ± 4.3	8.8 ± 5.6	0.77
	*p*	0.12	0.34	
Health Perception	T0	5.1 ± 3.3	8.5 ± 4.4	0.01 *
	T1	6.4 ± 2.9	7.7 (5.3–10.2)	0.08
	*p*	0.11	0.33	
Energy/Fatigue	T0	4.4 ± 2.8	7.2 (5.5–14.4)	0.003 *
	T1	4.6 ± 2.3	5.8 (4.8–6.9)	0.06
	*p*	0.73	0.03 *	
Role Physical Limitation	T0	3.0 (0.0–9.0)	6.0 (2.3–12.0)	0.17
	T1	0.0 (0.0–12.0)	6.5 (2.5–12.0)	0.09
	*p*	0.62	0.07	
Pain	T0	5.5 ± 3.3	6.1 (4.1–7.6)	0.51
	T1	6.2 ± 3.1	5.9 (4.1–7.4)	0.71
	*p*	0.28	1	
Sexual Functions	T0	52.8 (34.5–80.0)	18.3 (7.7–38.8)	0.003 *
	T1	53.2 (38.2–80.0)	14.0 (6.7–38.8)	0.004 *
	*p*	1	0.58	
Social Functions	T0	6.7 ± 3.4	7.5 (5.8–10.0)	0.21
	T1	8.5 (4.7–9.9)	8.0 (5.8–10.0)	0.38
	*p*	0.51	1	
Physical Health Distress	T0	5.1 ± 3.6	8.3 (5.2–11.0)	0.03 *
	T1	5.4 ± 3.3	7.1 ± 3.4	0.12
	*p*	0.5	1	
MSQOL Mental Composite	T0	46.2 ± 24.4	50.8 ± 12.8	0.46
	T1	50.9 ± 20.7	52.1 ± 14.0	0.84
	*p*	0.12	0.58	
Emotional Health Distress	T0	6.4 ± 4.5	10.0 (5.4–11.9)	0.06
	T1	6.9 ± 4.1	10.0 (5.4–12.6)	0.08
	*p*	0.47	1	
General Quality of Life	T0	7.6 ± 2.8	8.7 (7.2–10.3)	0.09
	T1	8.7 ± 3.2	8.7 (7.2–10.5)	0.41
	*p*	0.17	0.37	
Emotional Well-being	T0	13.3 ± 7.5	9.6 (7.7–12.0)	0.09
	T1	14.5 (11.3–20.0)	11.8 ± 3.1	0.06
	*p*	0.08	0.18	
Role Emotional Limitation	T0	7.9 (0.0–24.0)	10.4 (0.0–16.0)	0.7
	T1	11.9 (0.0–24.0)	12.4 /(1.0–14.5)	0.48
	*p*	0.25	0.06	
Cognitive Functions	T0	8.6 ± 4.1	10.5 (8.6–12.0)	0.61
	T1	8.0 ± 4.2	11.3 (9.0–12.0)	0.04 *
	*p*	0.35	0.17	

* *p* < 0.05. Data are presented as means ± standard deviations for normally distributed variables and medians (first and third quartiles) for non-normally distributed variables.

## Data Availability

Data are available to the corresponding author.

## Abbreviations

The following abbreviations are used in this manuscript:

MS	Multiple sclerosis
EG	Experimental group
CG	Control group
TNF	Tumor necrosis factor
WBV	Whole-body vibration
FMV	Focal muscle vibration
QoL	Quality of life
RRMS	Relapsing remitting MS
